# Magnetic Modes in Rare Earth Perovskites: A Magnetic-Field-Dependent Inelastic Light Scattering study

**DOI:** 10.1038/srep36859

**Published:** 2016-11-15

**Authors:** Surajit Saha, Bing-Chen Cao, M. Motapothula, Chun-Xiao Cong, Tarapada Sarkar, Amar Srivastava, Soumya Sarkar, Abhijeet Patra, Siddhartha Ghosh, Ariando, J. M. D. Coey, Ting Yu, T. Venkatesan

**Affiliations:** 1 NUSNNI-NanoCore, 5A Engineering Drive 1, National University of Singapore, 117411, Singapore; 2 Department of Physics, 2 Science Drive 3, National University of Singapore, 117542, Singapore; 3 Division of Physics and Applied Physics, School of Physical and Mathematical Sciences, Nanyang Technological University, 637371, Singapore; 4 NUS Graduate School for Integrative Sciences and Engineering, 28 Medical Drive, National University of Singapore, 117456, Singapore; 5 School of Physics and Centre for Research on Adaptive Nanostructures and Nanodevices, Trinity College, Dublin, Ireland; 6 Department of Electrical and Computer Engineering, National University of Singapore, 117576, Singapore

## Abstract

Here, we report the presence of defect-related states with magnetic degrees of freedom in crystals of LaAlO_3_ and several other rare-earth based perovskite oxides using inelastic light scattering (Raman spectroscopy) at low temperatures in applied magnetic fields of up to 9 T. Some of these states are at about 140 meV above the valence band maximum while others are mid-gap states at about 2.3 eV. No magnetic impurity could be detected in LaAlO_3_ by Proton-Induced X-ray Emission Spectroscopy. We, therefore, attribute the angular momentum-like states in LaAlO_3_ to cationic/anionic vacancies or anti-site defects. Comparison with the other rare earth perovskites leads to the empirical rule that the magnetic-field-sensitive transitions require planes of heavy elements (e.g. lanthanum) and oxygen without any other light cations in the same plane. These magnetic degrees of freedom in rare earth perovskites with useful dielectric properties may be tunable by appropriate defect engineering for magneto-optic applications.

Transition-metal oxides are important and interesting as they show a rich variety of properties such as high-k dielectric, ferroelectric, multiferroic, magnetic, colossal magneto-resistive and superconducting properties. Among them, wide band gap insulators with perovskite-based structures including LaAlO_3_, LaGaO_3_ and SrTiO_3_, are widely used as substrates for functional oxide thin films since they contain ions with closed electronic shells. Magnetically and optically these oxides are of little interest in of themselves, unless appropriate cations are incorporated to tune their electronic band structure as well as their magnetic and optical properties^1^. However, surfaces and interfaces may behave quite differently to the bulk. The 2-dimensional electron gas at the interface of two such non-magnetic, wide band gap oxides, polar LaAlO_3_ and non-polar SrTiO_3_ raises the fundamental question of what makes the interface conduct, that has led to numerous experiments exploring oxide interfaces^2^,^3^,^4^, using oxide thin films deposited on oxide substrates. It is intriguing that the interface not only conducts but may also show magnetism and superconductivity^5^,^6^,^7^,^8^. In other words, recent research has rekindled interest in this class of oxides and especially the two popular ones, LaAlO_3_ and SrTiO_3_, revealing a variety of novel interface phenomena. Very recently, it has been demonstrated that oxide-heterostructures with angular momentum states in rare earth perovskites lead to long-range oscillatory magnetic interaction across oxide interfaces via orbital coupling^9^. This magnetic interaction was explained by a long-range transmission of orbital magnetization mediated by the polar oxide barrier. Thus magnetic excitations are possible in specific polar oxide layers. Since these layers are available as single crystals, in order to identify the interface properties, it is important to have a good understanding of the behavior of the substrate crystals themselves. In this paper we demonstrate novel magnetic excitations via Raman spectroscopic studies in LaAlO_3_ and a variety of polar oxide substrates.

LaAlO_3_, a rare-earth based perovskite, has a dielectric constant of ~25 and a band gap of ~5.6 eV. This material is a promising gate insulator for advanced Metal-Oxide-Semiconductor (MOS) devices^10^,^11^,^12^. However, it has been reported to possess localized mid-gap states due to various types of defects — interstitials, vacancies, anti-sites, impurities — causing leakage current and it is important to understand the various defects and their dynamics both experimentally and theoretically^13^,^14^,^15^,^16^,^17^,^18^,^19^. On the other hand, lattice strain plays an important role on the defects and their dynamics and hence on the related physical properties^20^. LaAlO_3_ undergoes a structural phase transition from cubic to rhombohedral below 813 K (540 °C). The phase transition is associated with a rotation of the AlO_6_ octahedra that leads to lattice strain and the formation of twins and twin-boundaries in LaAlO_3_. Raman spectroscopy is a useful probe of the structural phase transitions in various ABO_3_ compounds, especially, LaAlO_3_. One of the early reports on Raman-active phonons of LaAlO_3_ at room temperature was by Scott^21^ showing four lattice vibrations at 33, 125, 155 (Ghost mode) and 487 cm^−1^. Recent reports^20^,^22^ show additional modes at 180, 203, and 470 cm^−1^ suggesting broken-symmetry in LaAlO_3_. On a similar note, resonant ultrasound spectroscopic study of LaAlO_3_ proposes freezing of atomic motions of La/Al at off-site positions away from their high symmetry positions^23^. In other words, undoped LaAlO_3_ is an interesting compound that inherently hosts a variety of defects, which may promise new functionalities with appropriate defect engineering.

Motivated by such possibilities, we have performed magnetic field-dependent Raman spectroscopic studies of LaAlO_3_ and related rare earth perovskites at low temperatures to understand the lattice phonons and the possible excitations involving defect states. In addition to the Raman modes, we have been able to observe several transitions at low temperatures that disappear at room temperature. The additional transitions are found to be magnetic field-sensitive showing Zeeman splitting or a magnetic field-dependent shift suggesting an unexpected magnetic degree of freedom in LaAlO_3_. We believe that inherent defects in LaAlO_3_ may lead to changes in the cationic/anionic oxidation states and the crystal electric field thus giving rise to spin/orbital degrees of freedom.

## Experimental Techniques

Single crystalline substrates of LaAlO_3_ (from CrysTec, GmbH, Germany, and MTI, USA) oriented along (100), (110), and (111) have been used to perform the experiments. We have not observed any gross difference in the quality of the substrates from these suppliers. We have also performed experiments on other rare-earth-based oxides, namely NdAlO_3_, NdGaO_3_, DyScO_3_, LSAT [(La_0.18_Sr_0.82_)(Al_0.56_Ta_0.41_)O_3_], LSAO (LaSrAlO_4_), and SrTiO_3_. In order to identify and quantify the contaminants in these single crystal substrates, we have conducted Particle Induced X-ray Emission (PIXE) measurements by using 2 MeV alpha particles and detecting the emitted X-rays using a Si(Li) detector that reveals the presence of no impurity above the instrumental resolution limit (~10 ppm).

Raman spectroscopic measurements at low temperatures as a function of magnetic field were performed using a WiTec Raman spectrometer coupled to an attoCube superconducting magnet. Further, temperature-dependent Raman spectra were recorded using a liquid nitrogen cooled Linkam stage. A 532 nm laser line was used as an excitation source while an 1800 g/mm grating was used to record the spectra. The spectra were also recorded using 488, 514.5, and 633 nm laser excitation in order to distinguish the Raman active and photoluminescent (PL) transitions. Raman spectra of LaAlO_3_ (and other oxides) were recorded using k//B configuration and both being perpendicular to the substrate surface.

## Results

Group theory predicts five (*A*_1g_ + 4*E*_g_) Raman active modes for 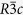 space group of rhombohedral LaAlO_3_. Experimentally these modes have been reportedly observed at 32 (*E*_g_), 123 (*A*_1g_), 152 (*E*_g_), 464 (*E*_g_), and 487 (*E*_g_) cm^−1^ while additional modes at 180 and 203 cm^−1^ were assigned to ghost modes^24^. On the other hand, a possibility of broken inversion symmetry led induced Raman activity has been discussed in ref. 22 which reassigns the modes to (5*E* + 3*A*_1_) symmetries. A comprehensive mode-assignment of the various optical phonons may be found in ref. 25. However, we have been able to observe various other modes which we believe are not optical phonons and were not reported earlier.

**Figure 1:**
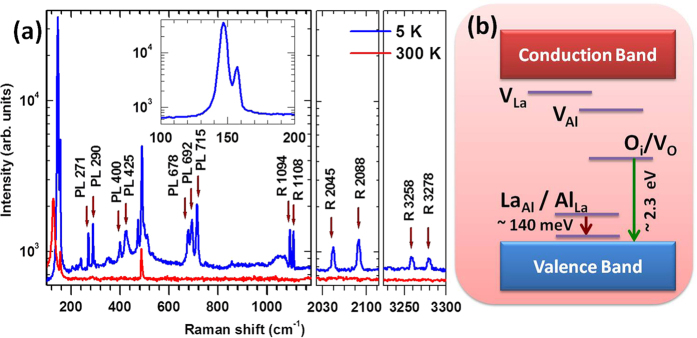
(**a**) The Raman spectra of LaAlO_3_ at 5 K and 300 K showing additional peaks, indicated by arrows (that show magnetic field dependence), arising at low temperatures which cannot be assigned to group theoretically predicted phonon modes. (**b**) A schematic diagram of the defect-induced mid-gap states which are responsible for the additional peaks, as discussed in the text.

**Figure 2:**
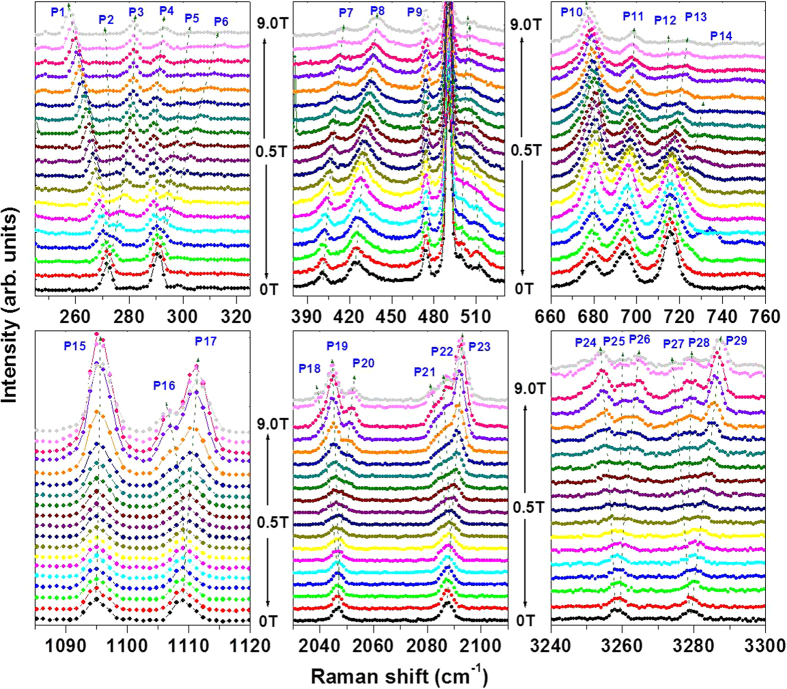
Magnetic-field dependence of all the new peaks seen at low temperatures. The peaks show Zeeman splitting or blue-shift under magnetic field indicating the presence of magnetic degrees of freedom (spin and/or orbital states). The split-peaks are labeled as P1 to P29.

**Figure 3:**
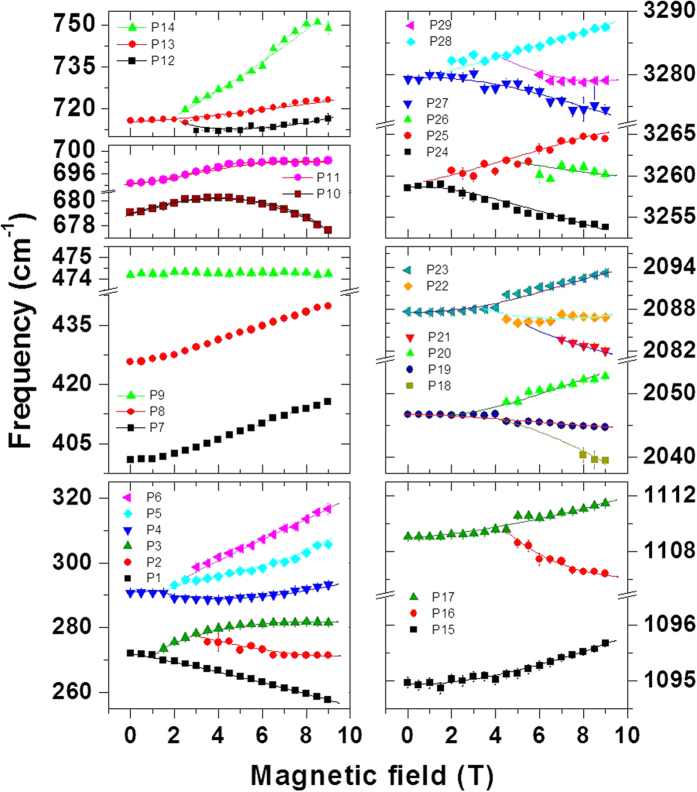
Frequency shift of the Zeeman-split peaks as a function of magnetic-field . Some of the peaks do not split but show blue-shift with increasing magnetic-field while the peaks P10 and P11 show unusual change in blue-to-red shift at higher fields which may arise due to a coupling between the levels.

Figure 1(a) shows Raman spectra of LaAlO_3_ at 300 and 5 K using the 532 nm laser excitation. We observe peaks at 125, 153, 210, 283, 470 and 488 cm^−1^ at 300 K most of which match with spectra reported earlier^20^,^21^,^22^,^26^. A review of Raman work on LaAlO_3_ suggests that while the peak at 125 cm^−1^ may be assigned to AlO_6_ octahedral vibration with *A*_1g_ symmetry, the peaks at 153, 470, and 488 cm^−1^ are AlO_6_ octahedral vibrations with *E*_g_ symmetry. Interestingly, several additional peaks appear at low temperatures. Nineteen different peaks have been observed at 5 K (ranging from 146 to 3278 cm^−1^ as shown in Table 1), many of which have not been reported before^20^,^21^,^22^,^26^. Using the other laser excitation lines, we have been able to assign these peaks either to Raman active (R) or PL transitions (PL) and their origin is summarized in Table 1. Further details are given in supplementary information (Figure S1). In addition to the Raman active phonons, LaAlO_3_ shows several new peaks at low temperatures which may be due to a resonance effect arising from the defect-induced mid-gap states near 2.2 eV (discussed later) upon matching with the excitation source at 532 nm. Notably these modes becomes weaker or invisible for the other excitation wavelengths. Figures 2 and 3 show the magnetic field-dependent changes of the Raman active and PL peaks. Notably, splitting of the peaks as a function of magnetic field occurs at about 2 T and 4 T indicating a possible role of defect complexes and their structural anisotropy giving rise to finite non-zero angular momentum which will be discussed later.

The interaction between an external magnetic field and the magnetic dipole moment associated with the total angular momentum of an ion or defect will lead to Zeeman splitting of the energy levels. While Zeeman splitting will start in most systems right above zero field several of the transitions observed show a field threshold above which the splitting begins which we do not understand at this point. Magnetic splitting of the peaks in the spectrum of LaAlO_3_ at low temperatures indicates the presence of some sort of magnetic moment. Splitting of a peak in to three components implies an angular momentum quantum number of ‘1’. One can estimate the Lande-*g* factor using the relation, Δ*E* = 2*gμ*_*B*_*B*, where Δ*E* stands for peak-peak splitting for a given applied magnetic-field *B* and *μ*_*B*_ is the Bohr magneton. For the PL peaks at 271 and 290 cm^−1^, *g* ≈ 3.0 ± 0.2, whereas for the Raman active peaks at 1094 and 1108 cm^−1^ and their higher orders, *g* ≈ 1.3 ± 0.2. However, it should be noted that the line-shifts are nonlinear in the applied field. It is intriguing to find the magnetic states in LaAlO_3_ where La^3+^, Al^3+^ and O^2−^ all have nonmagnetic, closed shell ground states. We believe they arise from defects, as discussed below.

**Figure 4:**
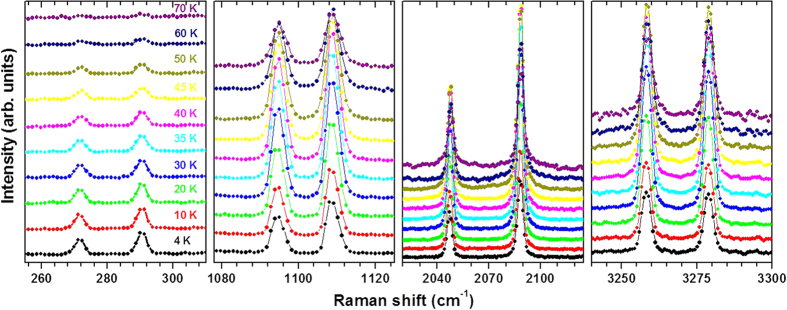
Raman spectra of the LaAlO_3_ as a function of temperature . The peaks get weaker at higher temperatures.

**Figure 5:**
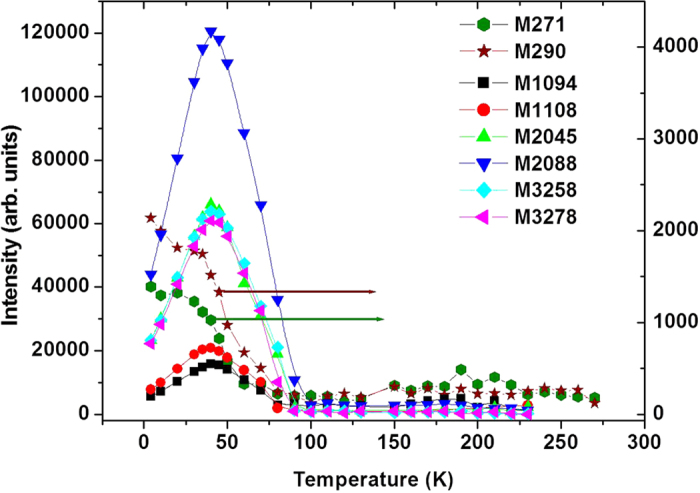
Intensity of the peaks (that are magnetic field sensitive) as a function of temperatures. The PL peaks at 271 and 290 cm^−1^ become very weak at high temperatures while the Raman peaks at 1094, 1108 cm^−1^ and their higher order peaks show an increase in intensity up to 40 K above which it drops dramatically.

Figure 4 shows the peaks that are magnetic field sensitive at different temperatures. It is important to note that the PL peaks at 271 and 290 cm^−1^ show a continuous drop in intensity as the temperature increases and practically disappearing above 70 K, as also can be seen in Fig. 5. However, the Raman-active peaks at 1094 and 1108 cm^−1^ and their higher orders (see supplementary for detail) initially show an increase in the intensity with temperature up to about ~40 K, but upon further increasing the temperature their intensity drops and they too become very weak above 100 K. The temperature dependence of the intensity of the peaks suggests that the PL peaks at 271 and 290 cm^−1^ involve transitions between the ground state and excited states (~2.3 eV) whereas the Raman active peaks near 1094 and 1108 cm^−1^ involve transitions between an intermediate state (that lies near ~40 K, i.e. ~30 cm^−1^ above the ground state) and excited states (situated near ~140 meV = 1120 cm^−1^). On looking at the temperature dependence, the PL transitions decay with increasing temperature as expected due to enhanced non-radiative transitions from phonon excitations. On the other hand, the Raman-active peaks show a bell shaped behavior as initially the transition increases with population of electrons in the intermediate level but as this population level increases further with increasing temperature it starts inhibiting the transition from the upper level.

## Discussion

Our experiments suggest the presence of defects with possible spin and/or orbital magnetic moments. Several recent reports of PL spectra in LaAlO_3_ have shown the presence of various types of defects via sharp or broad peaks below the bandgap. Kawabe *et al.*^27^ show the presence of a broad PL at ~2.5 eV attributed to oxygen vacancies whereas Chen *et al.*^18^ show the presence of two narrow peaks at 1.7 and 1.8 eV and a broad peak at 2.1 eV arising from the displacement of cations from their regular sites. Theoretically, it has been shown that in LaAlO_3_ Schottky-like defects that maintain charge neutrality are dominant under all conditions, besides oxygen vacancies and anti-site defects^19^. Luo *et al.* reported^16^ (using density functional theoretical [DFT] calculations) that charged and neutral oxygen interstitials give rise to three levels within about 2.2 eV above the valence band maximum (VBM). Recently, another DFT estimation by Choi *et al.*^28^ suggested that cationic vacancies, such as, Lanthanum vacancies with 0 or −3 charges give rise to a level at 290 meV above the VBM with a spin quantum number *S* = 3/2 and another level within 10 meV difference having spin *S* = 1/2 while aluminium vacancies with 0 or −3 charges give rise to a level at 690 meV above VBM and another at a separation of 20 meV with spin *S* = 3/2 and *S* = 1/2, respectively. Further, anti-site defects, such as La at Al-site (La_Al_) and Al at La-site (Al_La_) also give rise to additional levels: Al_La_ with 0/+1 charge introduces a level at 300 meV above VBM whereas La_Al_ with 0/+1 charge gives a level at 170 meV and +1/+2 charge at 40 meV. In other words, it has been proposed theoretically that various defects may introduce mid-gap states, some of which have spin character.

Our data provide direct evidence for the presence of angular momentum states in LaAlO_3_ as shown by the Zeeman splitting of peaks, which most likely arise from inherent defects. The data show a fair match with the energy levels estimated in refs 16 and 28, but the splitting is not generally consistent with S = 1/2 or S = 3/2. The triplet structure seen in most cases suggests J = 1, with g ≈ 3 for PL271 and PL290, g ≈ 4 for PL715 and g ≈ 1.3 for R2045, R2088, R3258 and R3278 peaks (see Figs 1 and 2). Further, the PL peaks start to split under magnetic field above 2 T while the Raman active peaks split at about 4 T. This suggests a possible role of two different types of defect-complexes having structural anisotropy, i.e., oxygen vacancies/interstitials forming a defect complex with La^3+^ that gives rise to the PL peaks while the cationic vacancies/anti-sites form another defect complex leading to the Raman active transitions. In presence of magnetic field, these defect complexes undergo structural transition at 2 and 4 T, respectively, thus giving rise to angular momentum states and the peak-splitting. In summary, our data demonstrate the idea of a magnetic excitation coupling to Raman active and photoluminescent transitions through defect formation where the spin-zero La^3+^ interacts with an oxygen vacancy/interstitial (or anti-site defect) to produce a defect complex with finite angular momentum. We think that in these rare-earth perovskites, in addition to such defects, the existence of an atomic plane with a high Z rare-earth ion and oxygen is a prerequisite to produce such angular momentum states. In the case of LaAlO_3_ it is possible that hybridization of the empty lanthanum ‘*d’* or ‘*f’* orbitals with the defect is somehow responsible for the effect.

This idea is consistent with our investigation of the magnetic-field dependence of the Raman spectra in other rare earth-based perovskite oxides. Data for NdAlO_3_, NdGaO_3_, and DyScO_3_ are shown in Figures S5 to S7 (see supplementary information). In Nd^3+^ and Dy^3+^ based compounds, we observe the angular momentum state transitions^29^,^30^,^31^,^32^,^33^ arising from *J* = 9/2 and *J* = 15/2 multiplets of Nd^3+^ and Dy^3+^, respectively, which exhibit Zeeman splitting under magnetic field (see a comparison in Figure S11). The contribution of the Nd^3+^/Dy^3+^ angular momentum states is higher than that of the defect complex in these oxides. However, in SrTiO_3_, where there is no heavy element in any plane with oxygen, we find no peak that exhibits any magnetic field dependence (Figure S8). Now what happens if a lighter cation is introduced in the atomic plane with the heavy element and oxygen? We have studied LSAT [(LaAlO_3_)_0.3_(Sr_2_AlTaO_6_)_0.7_] and LSAO [LaSrAlO_4_], and their spectra shown in Figures S9 and S10 do not indicate any magnetic field sensitive state. So the formation of the magnetic defect complex with lanthanum seems to be neutralized by the presence of a lighter cation in the same plane which requires further understanding.

We rule out a role of any magnetic impurity element such as Cr^3+^ and Fe^3+^ in the Al-based compounds^14^,^17^), especially in LSAT and LSAO and possibly in LaAlO_3_ as well. PIXE measurements showed that no impurities could be detected within the resolution limit (~10 ppm) in LaAlO_3_ (see Figure S12) and any of these substrates except SrTiO_3_ (~7 ppm of Cr and ~160 ppm of Fe; not shown in Figure). The fact that despite the presence of higher amounts of such magnetic impurities in STO, we do not see any magnetic-field sensitive excitation thus it suggests that native impurities are not responsible for these angular momentum states.

Therefore, the PL angular momentum state transitions observed near 271 and 290 cm^−1^ (in the absolute scale they are at ~2.3 eV) in LaAlO_3_ could be assigned to neutral oxygen interstitials or vacancies while the Raman active peaks near 1094 and 1108 cm^−1^ (~140 meV) may involve angular momentum states arising from anti-sites, especially La at Al-site (La_Al_) defects, and/or cationic vacancies. It should be noted that none of these magnetic-field-sensitive peaks which indicate the presence of magnetic degrees of freedom in LaAlO_3_ have ever been reported before^20^,^21^,^22^,^26^. Annealing of the LaAlO_3_ at ~900 °C in vacuum (*P*_*O*2_ = 1 × 10^−2^ mTorr) or oxygen rich environment (100 mTorr) for about 30 minutes does not have any significant effect on the magnetic-field sensitive peaks in the Raman spectrum (recorded using 532 nm laser line), demonstrating the stability and robustness of the defects present and suggesting possibilities for applications as optical magnetic-field sensor and light emitters. The key to the observation of the magnetic field sensitive states is the presence of a plane with a heavy element like the rare earth and oxygen. In SrTiO_3_ the absence of a plane of heavy elements means that we do not see any angular momentum state. Such angular momentum states in rare earth perovskites may be utilized to tailor the magnetic interactions across interfaces of oxide heterostructures for new emergent phenomena and device applications.

## Summary and Conclusion

Here, we have presented evidence for the presence of magnetic degrees of freedom in LaAlO_3_ crystals by performing magnetic-field-dependent Raman spectroscopy. In addition to the Raman active phonons, we have observed at low temperatures (below 100 K) several hitherto unknown peaks appearing due to Raman active transitions near 1094 and 1108 cm^−1^ (~140 meV) as well as a doublet luminescent transition near 2.3 eV (a few more weak transitions are also seen with similar energy). Under magnetic field, these additional peaks show Zeeman splitting or blue-shift thus indicating an association with orbital angular momentum states. We believe that these additional peaks arise from defects, possibly cationic/oxygen vacancies that trap electrons directly or populate states below the conduction band edge.

Our low temperature- and magnetic field-dependent Raman studies of LaAlO_3_ establish the presence of magnetic moments associated with defects in a purely diamagnetic material, which are inherent but robust. Engineering these defects and hence the angular momentum states may be a pathway to discover new phenomena^9^ and possible applications. We believe that our observations will motivate further theoretical and experimental studies to understand these complex oxides.

## Additional Information

**How to cite this article**: Saha, S. *et al.* Magnetic Modes in Rare Earth Perovskites: A Magnetic-Field-Dependent Inelastic Light Scattering Study. *Sci. Rep.* **6**, 36859; doi: 10.1038/srep36859 (2016).

**Publisher’s note:** Springer Nature remains neutral with regard to jurisdictional claims in published maps and institutional affiliations.

## Supplementary Material

Supplementary Information

## Figures and Tables

**Table 1:** Assignment of the peaks observed in the Raman spectrum of LaAlO_3_ at low temperature.

Peak Position (Raman shift: cm^−1^)	Mode assignment	Peak Position (Raman shift: cm^−1^)	Mode assignment
146 (R)	AlO_6_ vibration	678 (PL)	O_*i*_/V_O_
156 (R)	AlO_6_ vibration	692 (PL)	O_*i*_/V_O_
271 (PL)*	O_*i*_/V_O_	715 (PL)*	O_*i*_/V_O_
290 (PL)*	O_*i*_/V_O_	1094 (R)	La_Al_: anti-site/Cationic vacancy (1^st^ Order)
400 (PL)	O_*i*_/V_O_	1108 (R)*	La_Al_: anti-site/Cationic vacancy (1^st^ Order)
425 (PL)	O_*i*_/V_O_	2045 (R)*	La_Al_: anti-site/Cationic vacancy (2^nd^ Order)
473 (R)	AlO_6_ vibration	2088 (R)*	La_Al_: anti-site/Cationic vacancy (2^nd^ Order)
490 (R)	AlO_6_ vibration	3258 (R)*	La_Al_: anti-site/Cationic vacancy (3^rd^ Order)
500 (PL)	O_*i*_/V_O_	3278 (R)*	La_Al_: anti-site/Cationic vacancy (3^rd^ Order)
511 (PL)	O_*i*_/V_O_	R: Raman active; PL: Photoluminescence *Shows magnetic splitting	
