# Supplementary material for: Magnetic Modes in Rare Earth Perovskites: A Magnetic-Field-Dependent Inelastic Light Scattering study
Source: Sci Rep. 2016 Nov 15;6:36859. doi: 10.1038/srep36859 (PMC5109287; doi:10.1038/srep36859)
Supplement: Supplementary Information [file srep36859-s1.doc]

**Supplementary Information (Figures)**

**Magnetic Modes in Rare Earth Perovskites:**

**A Magnetic-Field-Dependent Inelastic Light Scattering study**

Surajit Saha,^1,2,‡,*^ Bing-Chen Cao,^3^ M. Motapothula,^1,2^ Chun-Xiao Cong,^3^ Tarapada Sarkar,^1,2^ Amar Srivastava,^1,2^ Soumya Sarkar,^1,4^ Abhijeet Patra,^1,4^ Siddhartha Ghosh,^1^ Ariando,^1,2^

J. M. D. Coey,^1,5^ Ting Yu,^3^ and T. Venkatesan^1,2,4,6,*^

^1^NUSNNI-NanoCore, 5A Engineering Drive 1, National University of Singapore, Singapore 117411

^2^Department of Physics, 2 Science Drive 3, National University of Singapore, Singapore 117542

^3^Division of Physics and Applied Physics, School of Physical and Mathematical Sciences, Nanyang Technological University, Singapore 637371

^4^NUS Graduate School for Integrative Sciences and Engineering, 28 Medical Drive, National University of Singapore, Singapore 117456

^5^School of Physics and Centre for Research on Adaptive Nanostructures and Nanodevices, Trinity College, Dublin, Ireland

^6^Department of Electrical and Computer Engineering, National University of Singapore, Singapore 117576

To get a clear picture of the origin of the peaks at low temperatures, we have recorded the Raman spectra of LaAlO_3_ (at 80 K and above) using several excitation energies (wavelengths), e.g. (λ_exc_ =) 488, 514.5, and 633 nm, in addition to the one at 532 nm. Figure S1 shows a comparison of the Raman spectra at 80 K at various excitation wavelengths suggesting that the peaks at 146, 156, 473, and 490 cm^-1^ are present irrespective of the excitation wavelength thus confirming their Raman activity. It is important to note that the spectra recorded using (λ_exc_ =) 488 and 633 nm laser lines do not show such a large number of peaks as seen in the case of λ_exc_ = 532 nm. The peaks at 271 and 290 cm^-1^ for λ_exc_ = 532 nm are found to be shifted to ~ 910 and 930 cm^-1^ for λ_exc_ = 514.5 nm thus suggesting their photoluminescent (PL) origin. The peaks at 400, 425, 500, 511, 678, 692, and 715 cm^-1^, seen for λ_exc_ = 532 nm, could not be clearly seen upon exciting with other laser lines. Notably, two peaks are seen at 1115 and 1140 cm^-1^ for λ_exc_ = 514.5 nm. It is not clear if the origin of these two peaks is the same as that of the two peaks at 1094 and 1108 cm^-1^ seen for λ_exc_ = 532 nm because their intensities are weaker for λ_exc_ = 514.5 nm and importantly their positions are slightly shifted for the two different laser excitation sources. As discussed in the text, LaAlO_3_ is known to possess various types of defects: cationic and oxygen vacancies, interstitials, and anti-site defects. In Figure 1(b), we show the possible mid-gap states arising from these various types of defects [16, 18, 19]. As suggested by DFT calculations reported previously [16, 19, 28], there are several mid-gap states (at around 2.3 eV) arising from various defects (as discussed in the main text). Hence, the possibility of a resonant excitation [22] arising from the energy-match of the exciting laser line (2.33 eV) with any of the mid-gap states (arising from defects) [16, 19, 28] cannot be ruled out. This potential resonance phenomena may as well explain the difference in the intensity of the peaks near 1094 and 1108 cm^-1^ for λ_exc_ = 532 nm with those at 1115 and 1140 cm^-1^ for λ_exc_ = 514.5 nm. On the other hand, additional doublet-peaks have also been seen for λ_exc_ = 532 nm at 2045, 2088, 3258, and 3278 cm^-1^ which may be attributed to the higher order modes of the doublets at 1094 and 1108 cm^-1^. Though the actual origin of these doublets are not clear at present, their intensity ratio (See Figure S2) and especially the similarity in their temperature dependence (See Figure 5) possibly indicate that they are higher order modes of the doublets at 1094 and 1108 cm^-1^. The 2^nd^ and 3^rd^ order peaks are not seen with other laser excitations possibly due to off-resonance. To be noted that under magnetic field the higher order peaks (at 2045, 2088, 3258 and 3278 cm^-1^) clearly split into three components whereas the first order peaks (at 1094 and 1108 cm^-1^) do not. This may be due to the fact that the spectral resolution is not sufficient to see splitting of the first order peaks. Nonetheless, a clear broadening of the linewidth of the first order peaks (at 1094 and 1108 cm^-1^), as shown in Figure S3, is a testimony of the increasing separation of their nearly-degenerate and unresolved components.

Table S1: Summary of the wavelength dependence of the Raman spectrum of LaAlO_3_

| Peak Position  (in cm^-1^) with  λ_exc_ = 532 nm | Peak Position  (in cm^-1^) with  λ_exc_ = 514.5 nm | Peak Position  (in cm^-1^) with  λ_exc_ = 488 nm | Peak Position  (in cm^-1^) with  λ_exc_ = 633 nm | Comment |
| --- | --- | --- | --- | --- |
| 146 | 146 | 146 | 146 | Raman active |
| 156 | 156 | 156 | 156 | Raman active |
| 271 | 910 | Not seen | Not seen | Photoluminescence |
| 290 | 930 | Not seen | Not seen | Photoluminescence |
| 400 | Not seen | Not seen | Not seen | - |
| 425 | Not seen | Not seen | Not seen | - |
| 473 | 473 | 473 | 473 | Raman active |
| 490 | 490 | 490 | 490 | Raman active |
| 500 | Not seen | Not seen | Not seen | - |
| 511 | Not seen | Not seen | Not seen | - |
| 678 | Not seen | Not seen | Not seen | - |
| 692 | Not seen | Not seen | Not seen | - |
| 715 | Not seen | Not seen | Not seen | - |
| 1094 | 1115 | Not seen | Not seen | Raman active (1^st^ Order) |
| 1108 | 1140 | Not seen | Not seen | Raman active (1^st^ Order) |
| 2045 | Not seen | Not seen | Not seen | Raman active (2^nd^ Order) |
| 2088 | Not seen | Not seen | Not seen | Raman active (2^nd^ Order) |
| 3258 | Not seen | Not seen | Not seen | Raman active (3^rd^ Order) |
| 3278 | Not seen | Not seen | Not seen | Raman active (3^rd^ Order) |


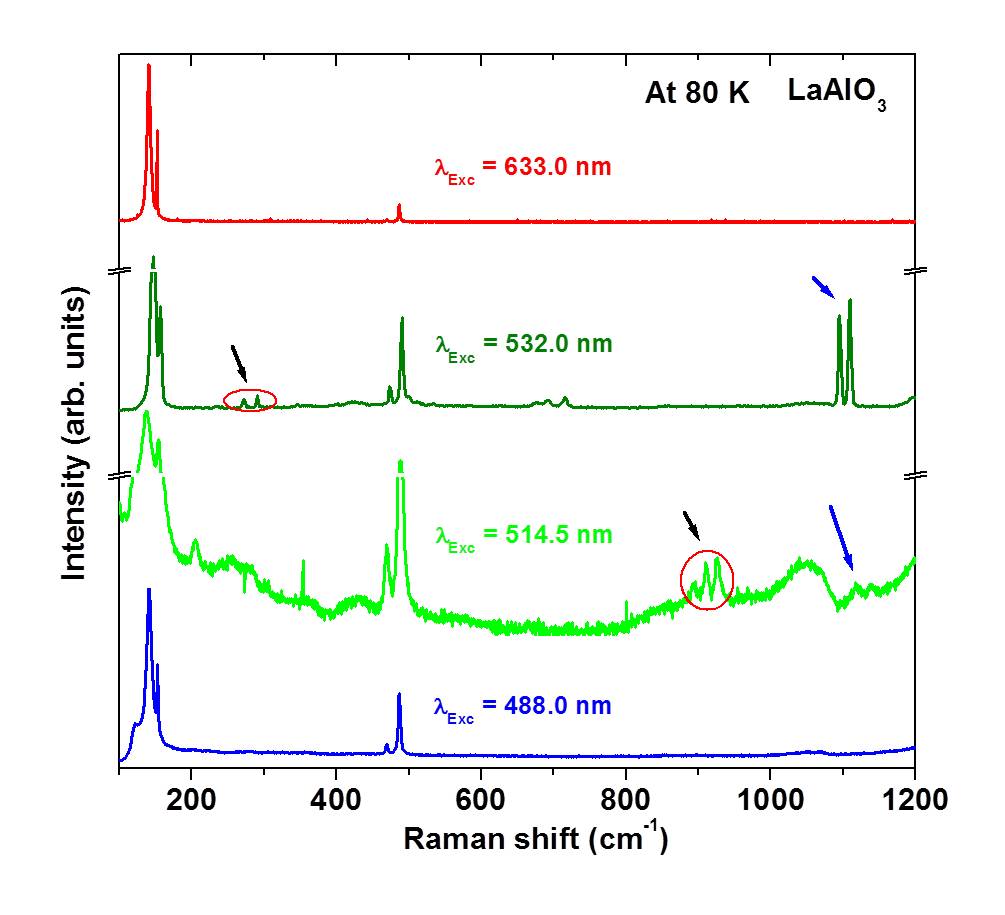


**Figure S1:** Raman spectra of LaAlO_3_ at 80 K recorded using (λ_exc_) 488, 514.5, 532, and 633 nm laser line. Raman active phonons of LaAlO3 are observed at the same positions irrespective of the excitation source. The spectrum with λ_exc_ = 532 nm has peaks at 271 and 290 cm^-1^ which are shifted to 910 and 930 cm^-1^ in spectrum with λ_exc_ = 514.5 nm confirming their PL origin. However, the peaks at 1094 and 1108 cm^-1^ in spectrum with λ_exc_ = 532 nm are slightly red-shifted in the spectrum with λ_exc_ = 514.5 nm possibly suggesting their resonant Raman activity.


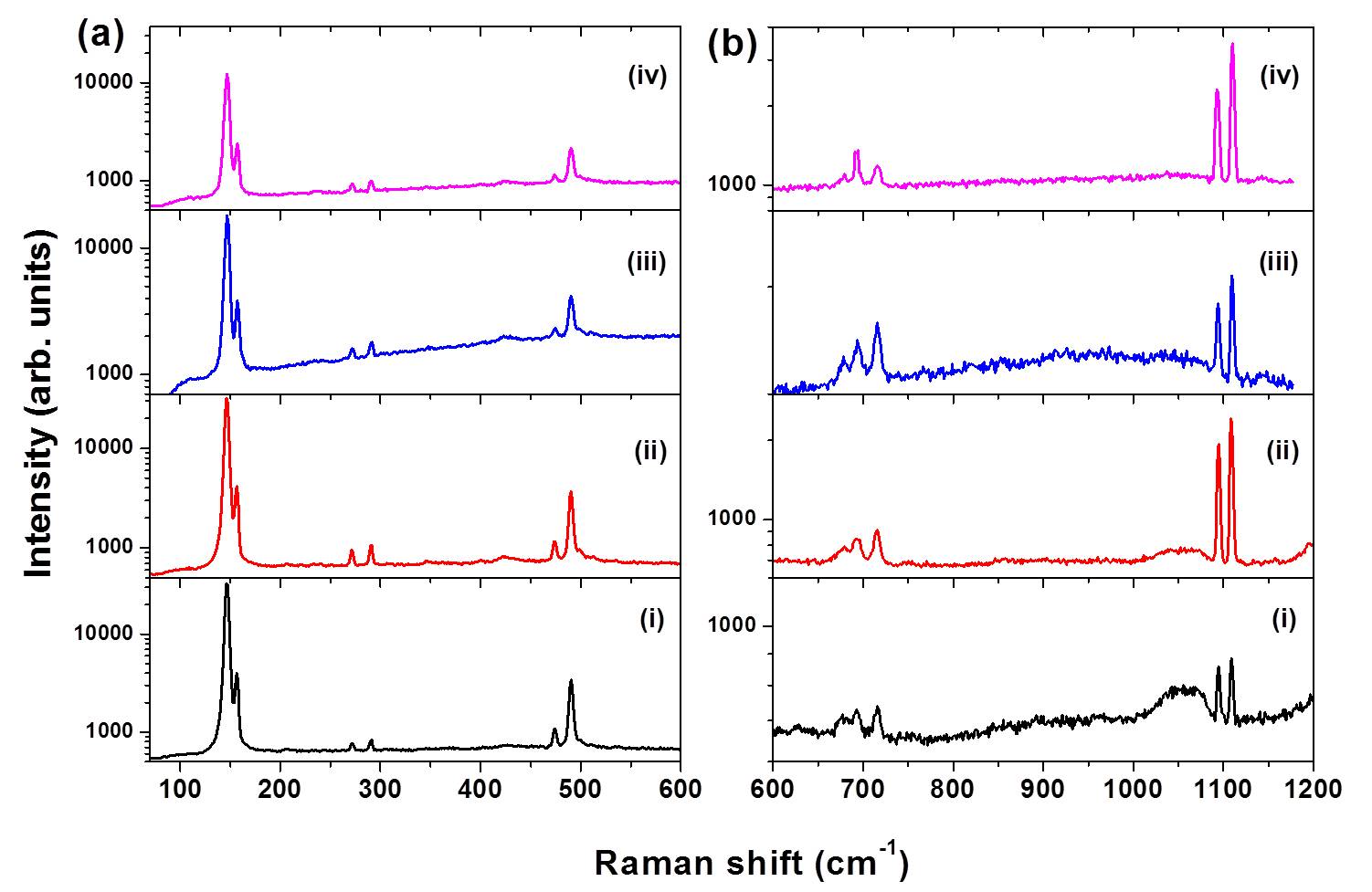


**Figure S2:** Raman spectrum of LaAlO_3_ substrate at 5 K oriented along (100) (i) obtained from Latech, Singapore, (ii) obtained from CrysTec, Germany, (iii) but recorded on the cleaved-plane after cleaving along the twinning of the substrate from CrysTec, Germany, and (iv) obtained from MTI, USA. Notably, all the samples show all the modes under discussion emphasizing the reproducibility of the modes.


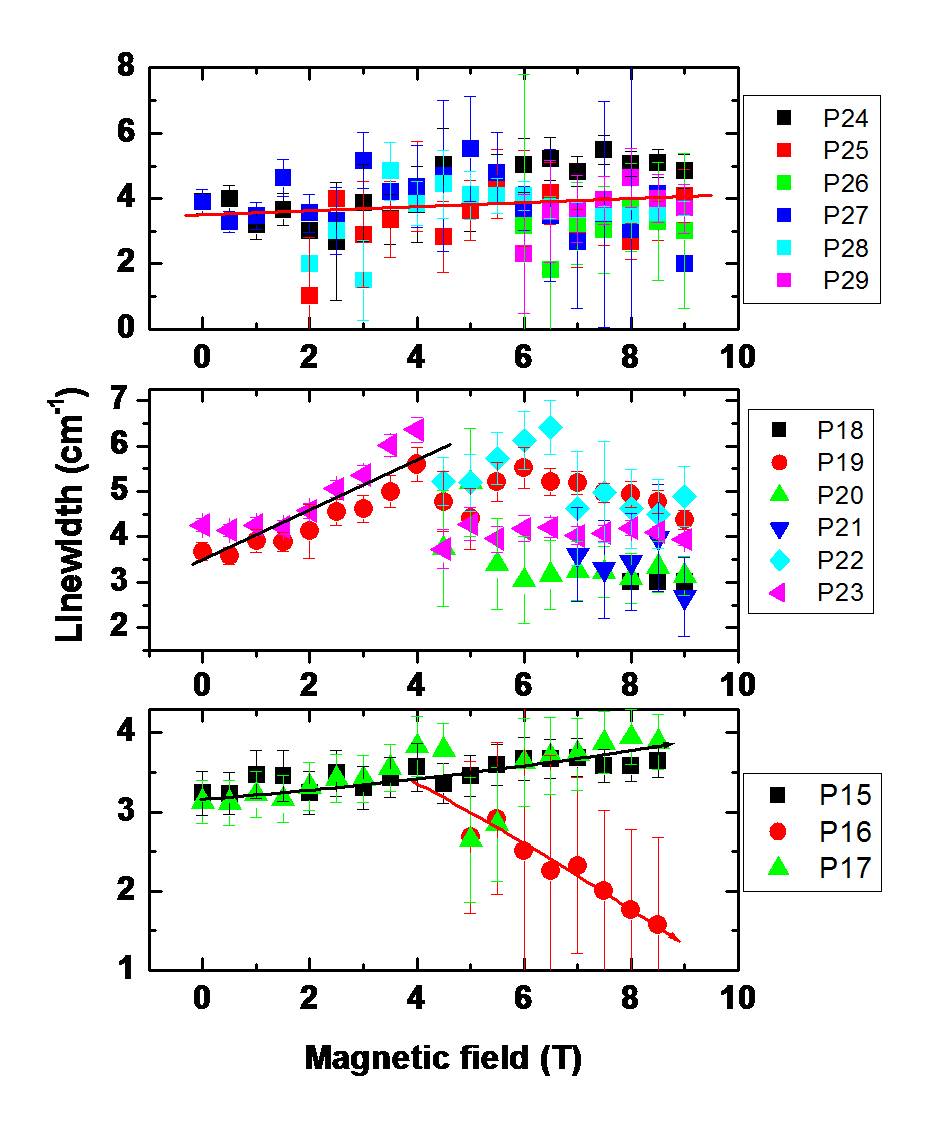


**Figure S3:** Linewidth of the Raman lines R1094, R1108, R2045, R2088, R3258 and R3278 and their split-bands at 5 K as a function of magnetic field. A clear increase in the linewidth is seen with increasing magnetic field for R1094, R1108, R2045 and R2088 lines.


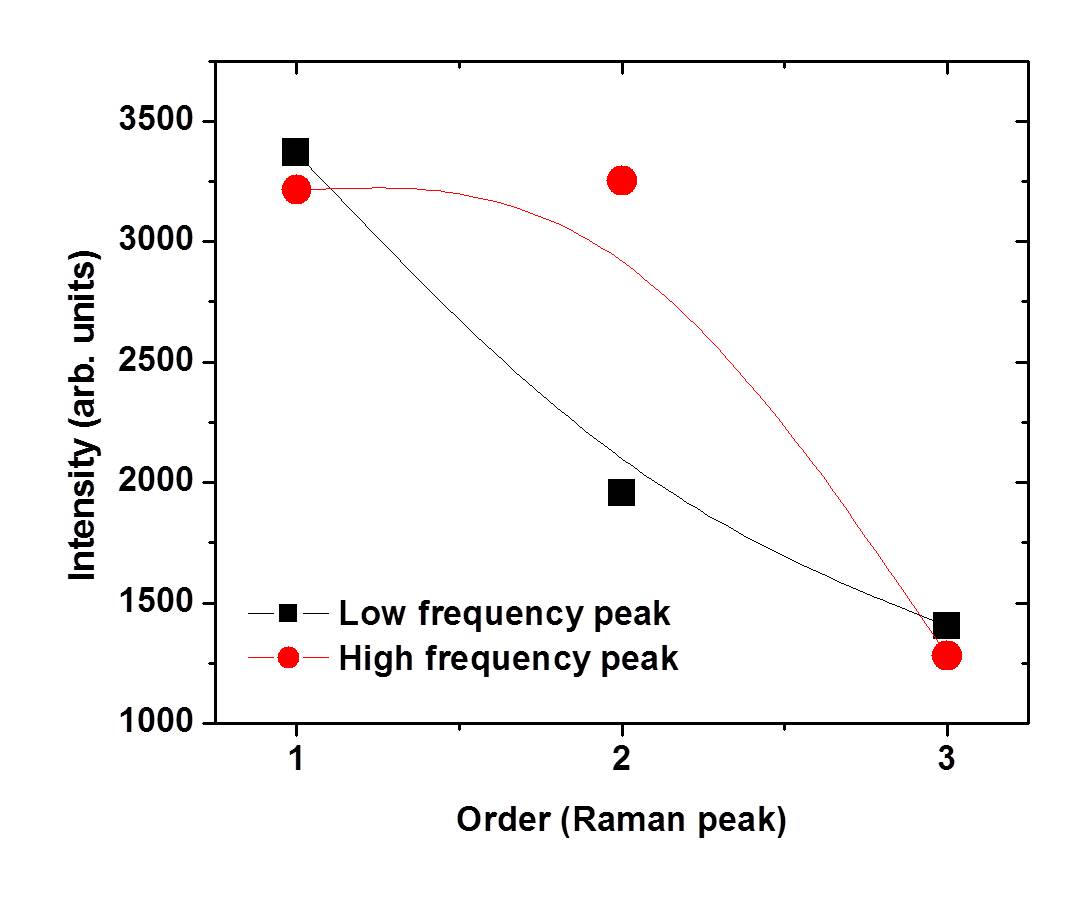


**Figure S4:** Intensity of the Raman active transitions at 1094 (black square) and 1108 cm^-1^ (red circle) and their second and third order peaks. A gradual decrease in their intensity and their frequency-values indicate them to be the first and higher order peaks.


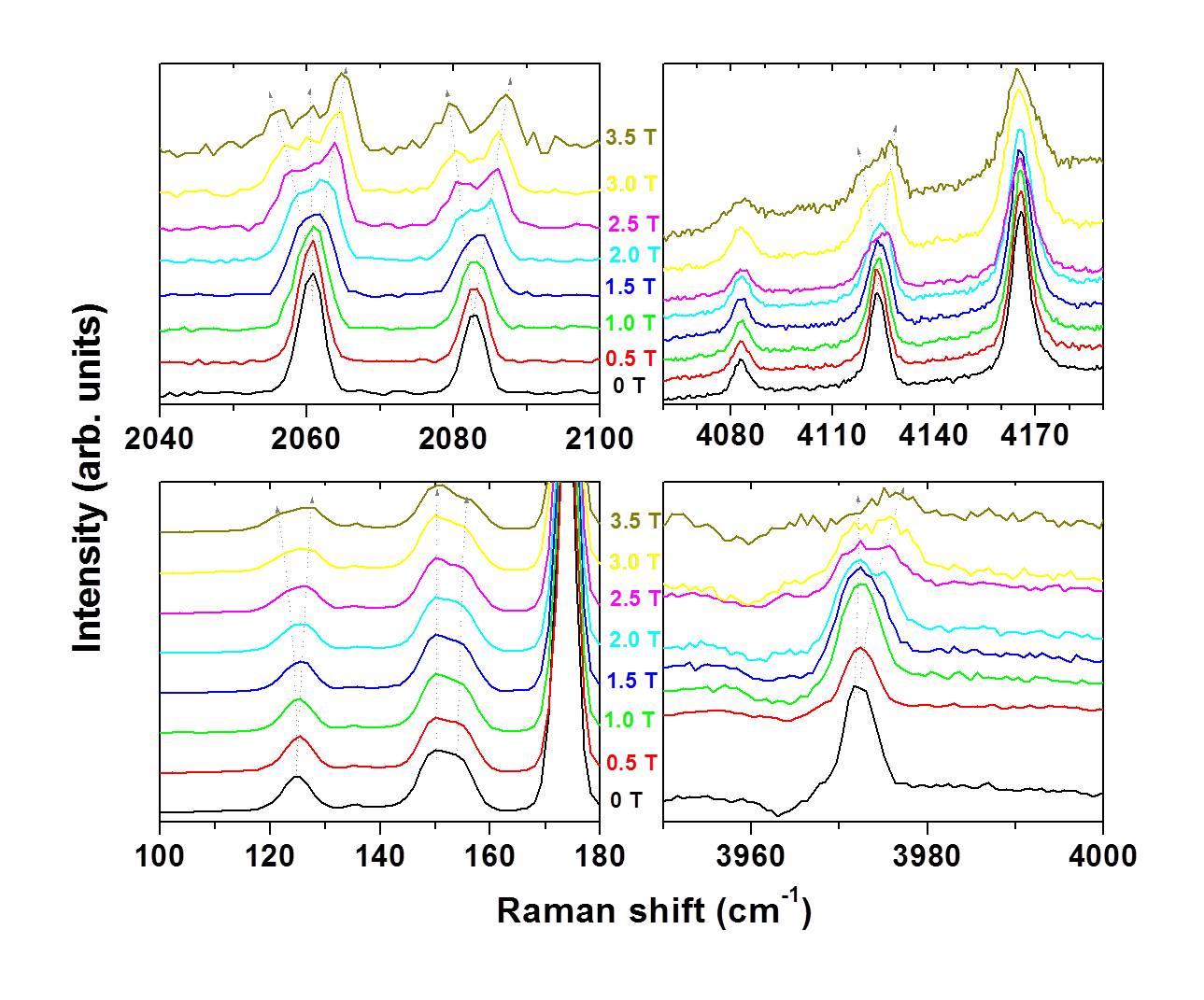


**Figure S5:** Magnetic field dependence of the Raman spectrum of NdAlO_3_ at 5 K. The peaks show Zeeman splitting with increasing magnetic-field. These peaks correspond to the transitions between the angular momentum states of J=9/2 multiplets of Nd^3+^. The spectra are recorded using λ_exc_ = 532 nm.


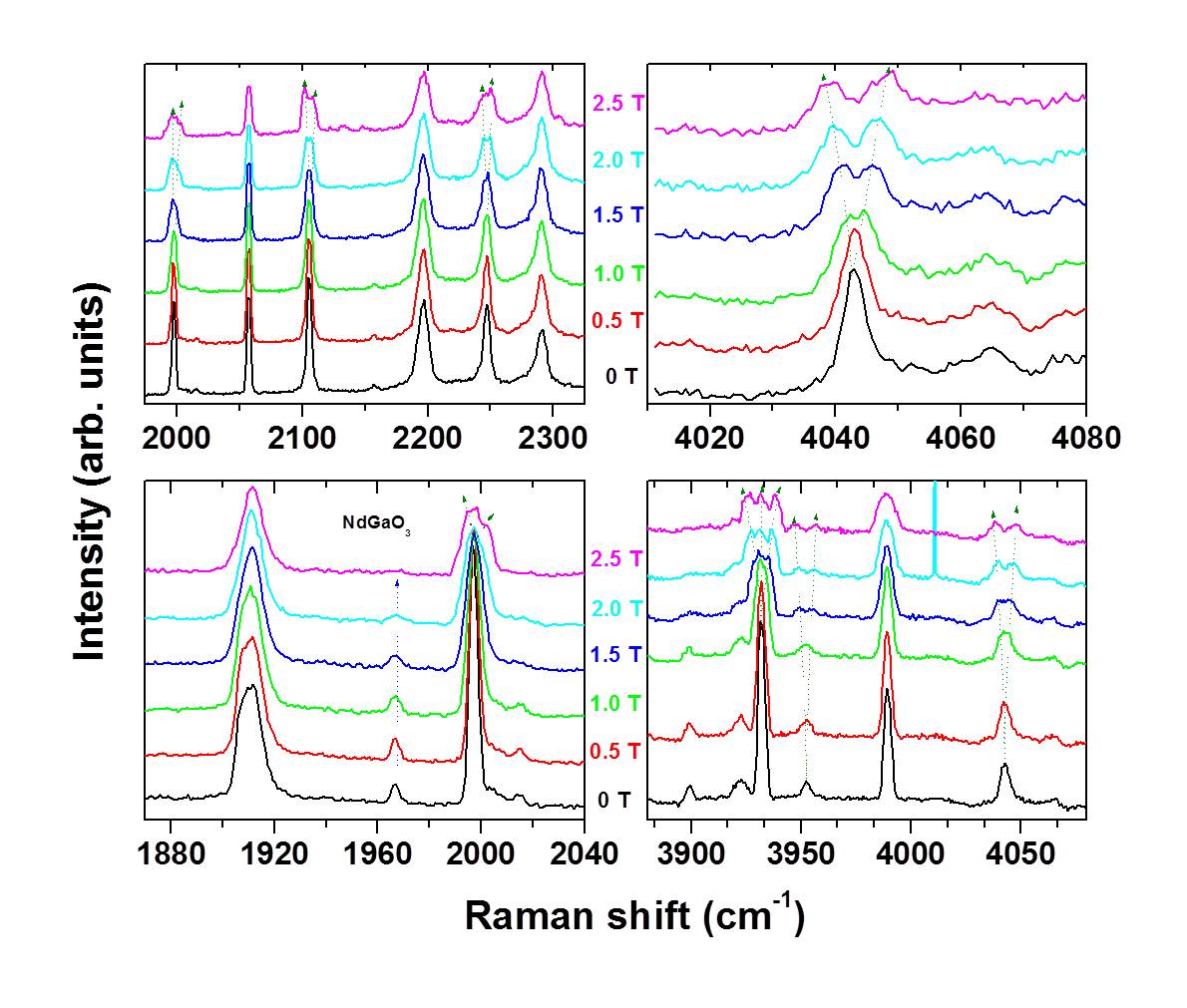


**Figure S6:** Magnetic field dependence of the PL transitions of NdGaO_3_ showing Zeeman splitting. The peaks originate from the transitions between angular momentum levels of J=9/2 multiplets of Nd^3+^. The spectra are recorded using a 532 nm laser line.


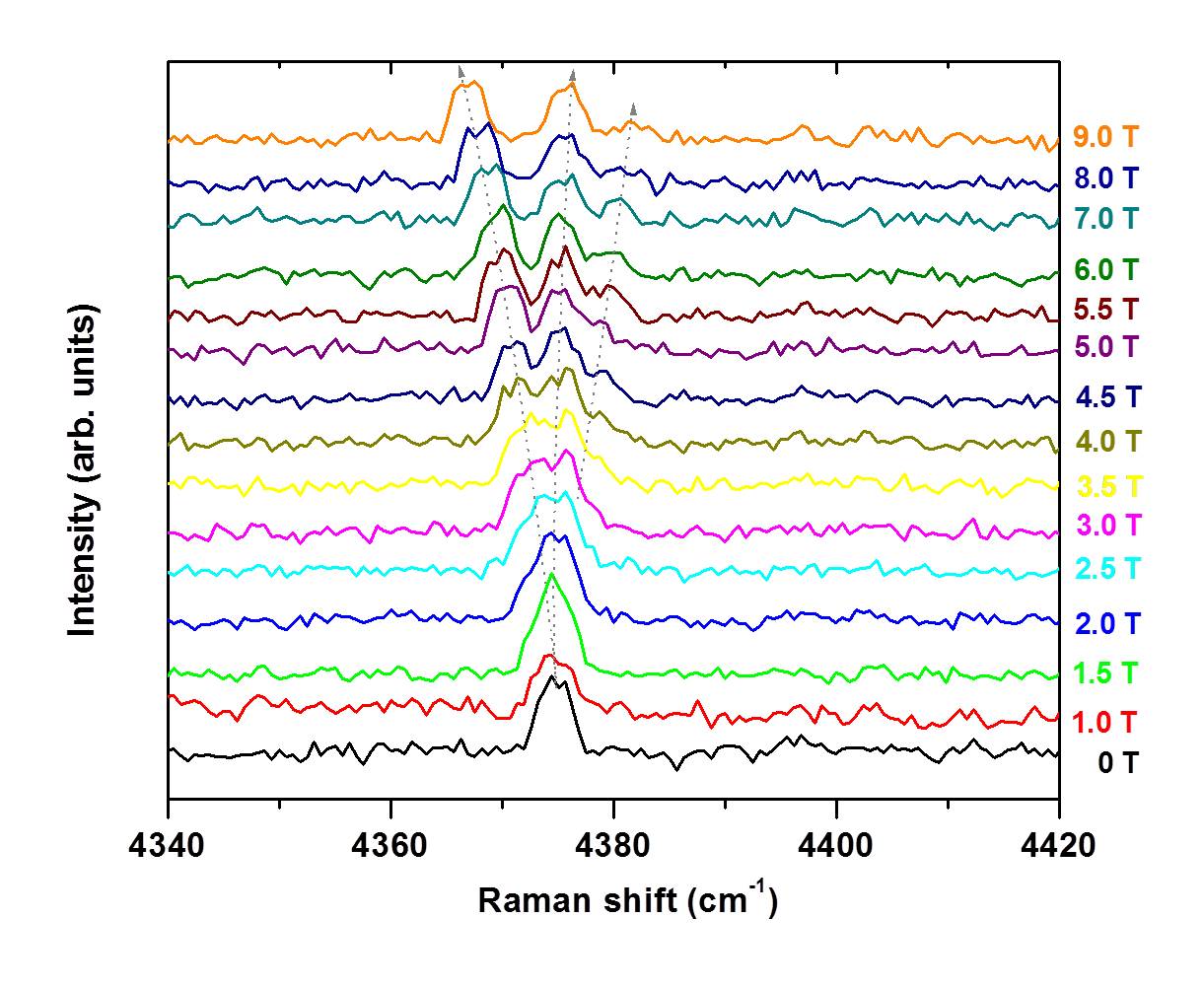


**Figure S7:** Zeeman splitting of a PL transition of DyScO_3_ involving angular momentum states of J=15/2 multiplets of Dy^3+^. The spectra are recorded using λ_exc_ = 532 nm.


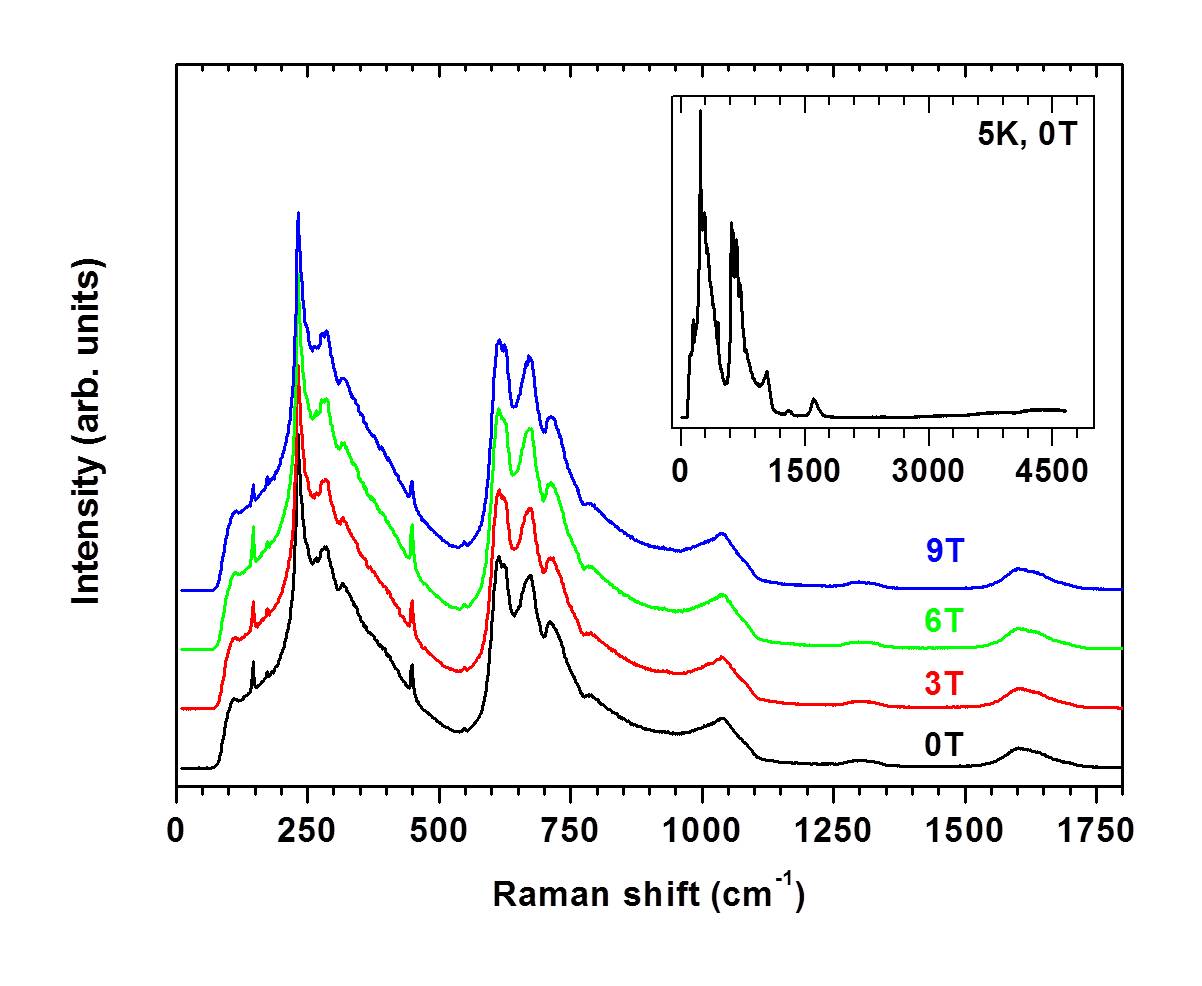


**Figure S8:** Raman spectra of SrTiO_3_ at 5 K as a function of magnetic field showing no magnetic field dependence.


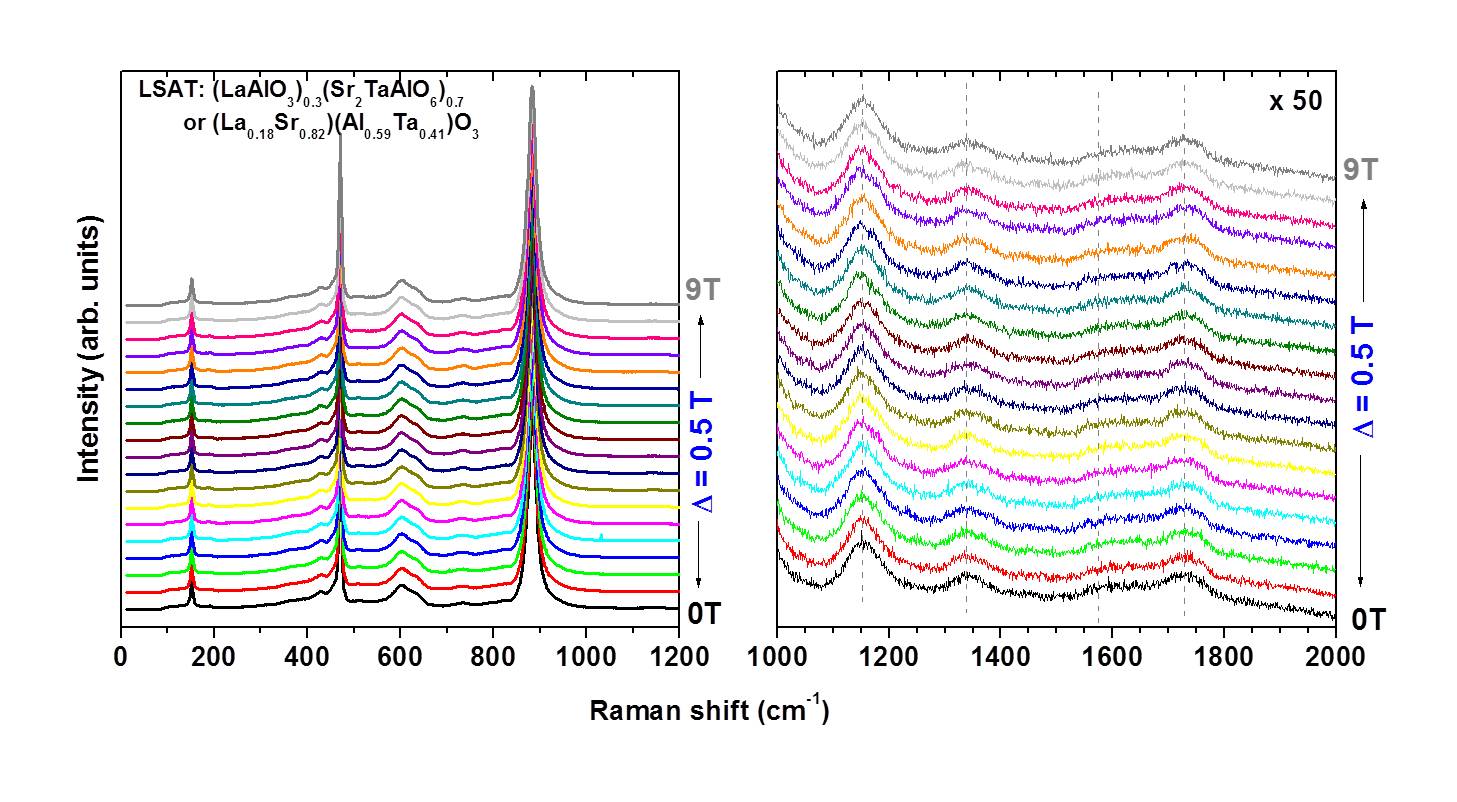


**Figure S9:** Magnetic field dependent Raman spectra of LSAT at 5 K. No Zeeman splitting has been observed.


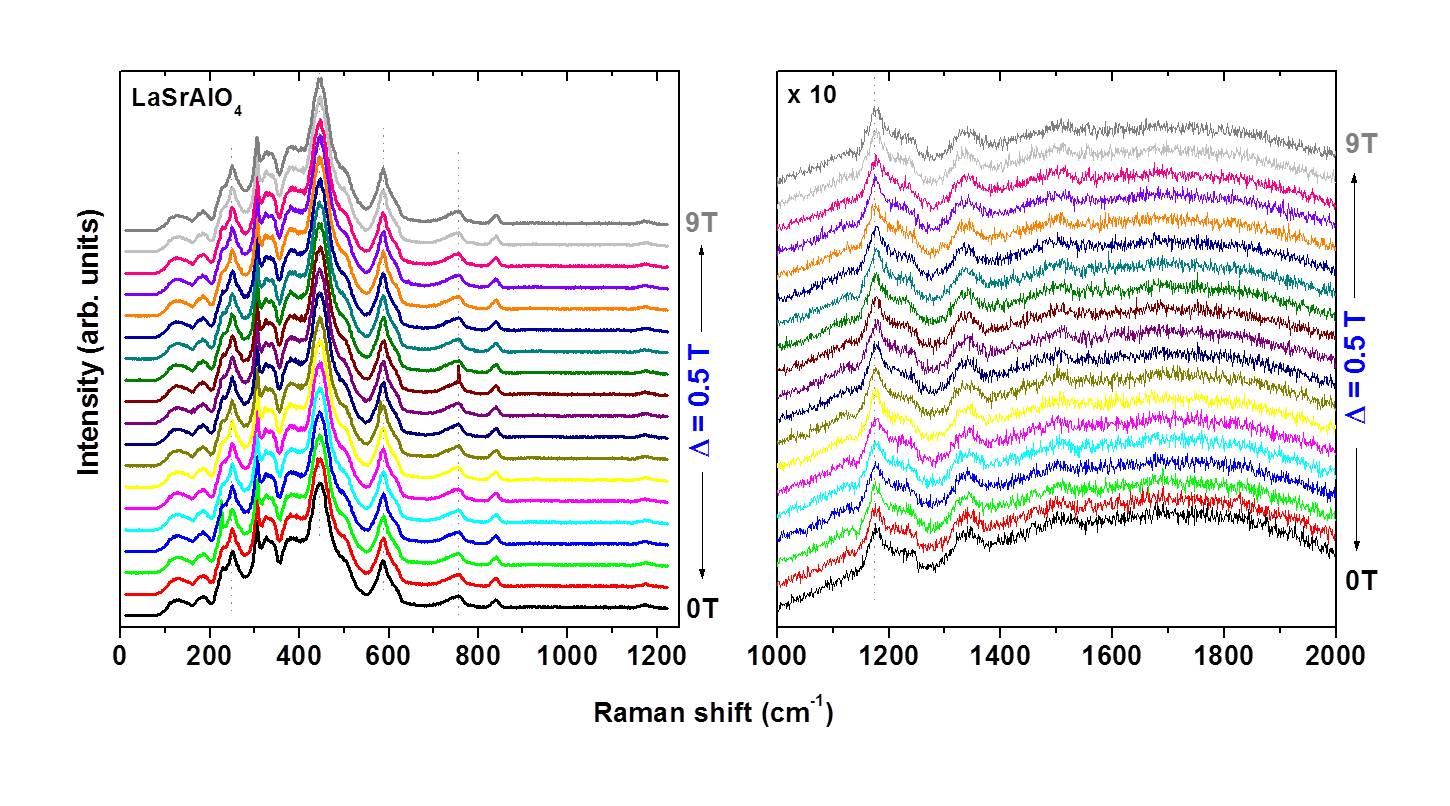


**Figure S10:** Magnetic field dependent Raman spectra of LaSrAlO_4_ at 5 K. No Zeeman splitting has been observed.


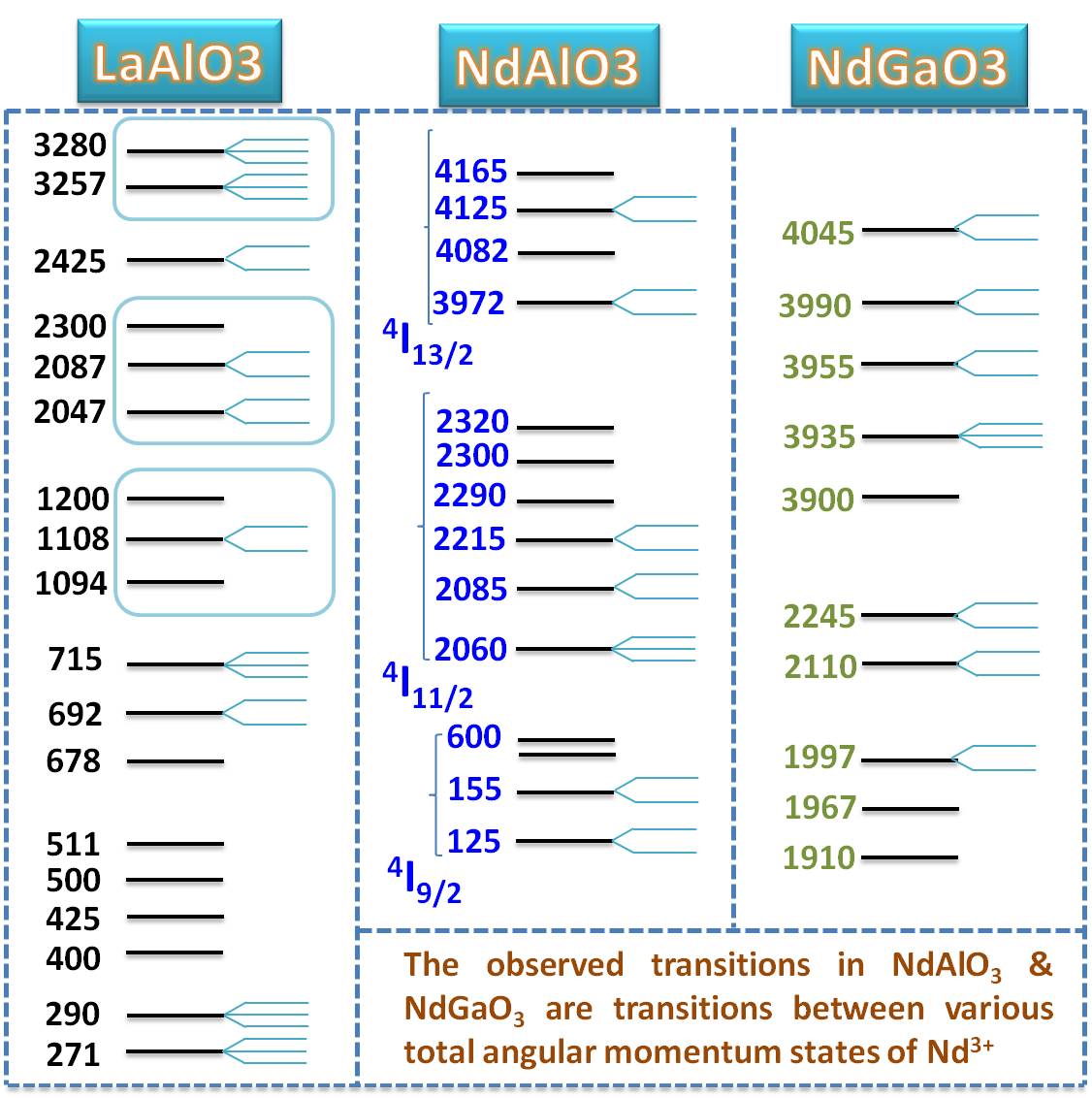


**Figure S11:** A schematic table summarizing the magnetic field sensitive levels of LaAlO_3_, NdAlO_3_ and NdGaO_3_.


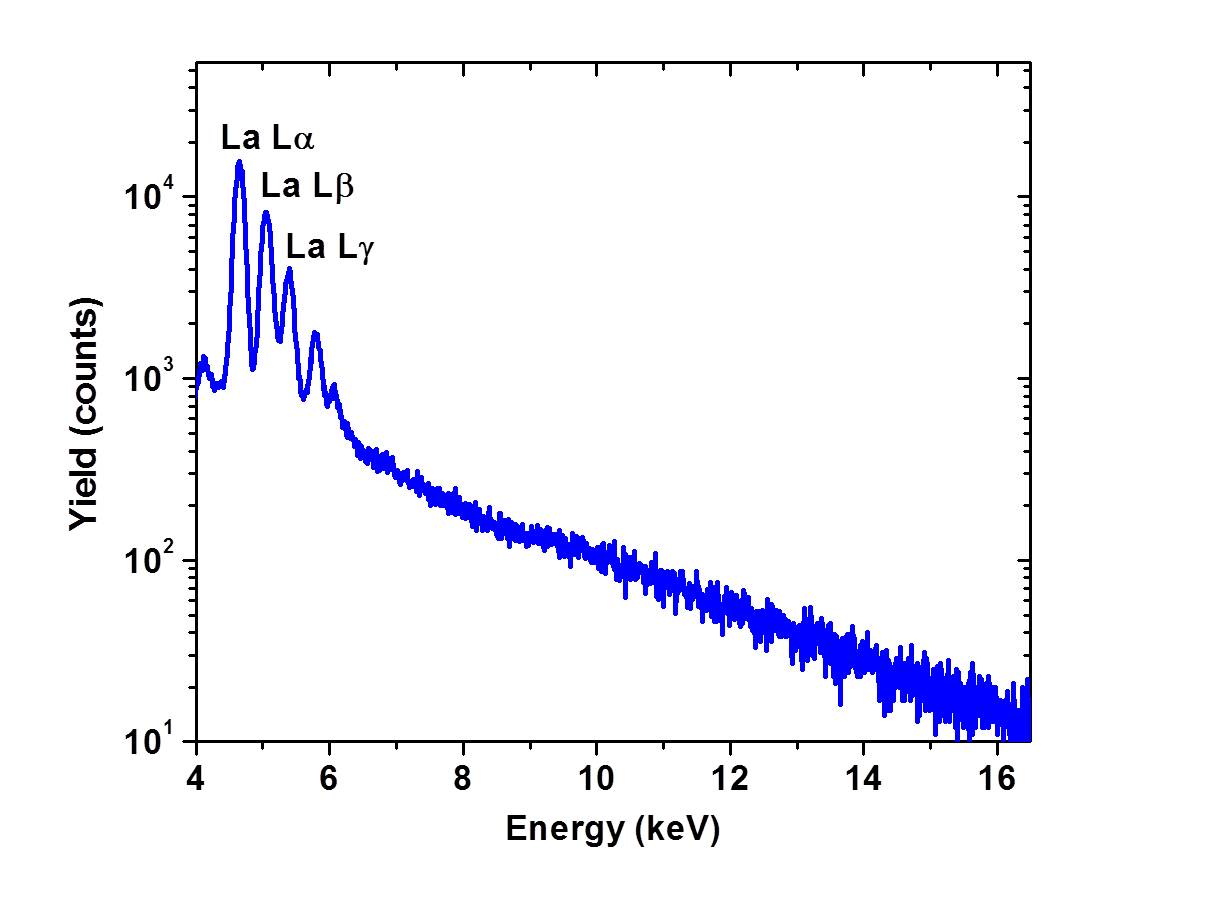


**Figure S12:** Proton-Induced X-ray Emission spectrum of LaAlO_3_ recorded using 2 MeV alpha-particles.
